# Cross-cultural adaptation of the short-form condom attitude scale: validity assessment in a sub-sample of rural-to-urban migrant workers in Bangladesh

**DOI:** 10.1186/1471-2458-13-240

**Published:** 2013-03-19

**Authors:** Tapash Roy, Claire Anderson, Catrin Evans, Mohammad Shafiqur Rahman, Mosiur Rahman

**Affiliations:** 1 Division of Social Research in Medicines and Health and School of Pharmacy, The University of Nottingham, Nottingham, UK; 2 School of Nursing, Midwifery and Physiotherapy, The University of Nottingham, Nottingham, UK; 3 Department of Statistical Sciences, University College London, London, UK; 4 Institute of Statistical Research and Training, University of Dhaka, Dhaka, Bangladesh; 5 BRAC Health, Nutrition and Population Programme, BRAC Centre (16th Floor), 75 Mohakhali, Dhaka 1212, Bangladesh

**Keywords:** Condom attitudes, Scale development, Vulnerable populations, Bangladesh

## Abstract

**Background:**

The reliable and valid measurement of attitudes towards condom use are essential to assist efforts to design population specific interventions aimed at promoting positive attitude towards, and increased use of condoms. Although several studies, mostly in English speaking western world, have demonstrated the utility of condom attitude scales, very limited culturally relevant condom attitude measures have been developed till to date. We have developed a scale and evaluated its psychometric properties in a sub-sample of rural-to-urban migrant workers in Bangladesh.

**Methods:**

This paper reports mostly on cross-sectional survey components of a mixed methods sexual health research in Bangladesh. The survey sample (n = 878) comprised rural-to-urban migrant taxi drivers (n = 437) and restaurant workers (n = 441) in Dhaka (aged 18–35 years). The study also involved focus group sessions with same populations to establish the content validity and cultural equivalency of the scale. The current scale was administered with a large sexual health survey questionnaire and consisted of 10 items. Quantitative and qualitative data were assessed with statistical and thematic analysis, respectively, and then presented.

**Results:**

The participants found the scale simple and easy to understand and use. The internal consistency (*α*) of the scale was 0.89 with high construct validity (the first component accounted for about 52% of variance and second component about 20% of the total variance with an Eigen-value for both factors greater than one). The test-retest reliability (repeatability) was also found satisfactory with high inter-item correlations (the majority of the intra-class correlation coefficient values was above 2 and was significant for all items on the scale, p < 0.001). The 2-week repeatability assessed by the Pearson product–moment correlation coefficient was 0.75.

**Conclusion:**

The results indicated that Bengali version of the scale have good metric properties for assessing attitudes toward condom use. Validated scale is a short, simple and reliable instrument for measuring attitudes towards condom use in vulnerable populations like current study sample. This culturally-customized scale can be used to monitor the progress of condom uptake and promotion activities in Bangladesh or similar settings.

## Background

It has been suggested that consistent use of condoms is likely to reduce the risk of the transmission of human immunodeficiency virus (HIV), as well as other sexually transmitted infections (STIs) [1]. Over the past several decades, public health agencies have implemented a variety of campaigns to increase the use of condoms, especially among vulnerable populations for HIV. However, in spite of such recognition and efforts, little is known about use of and attitudes towards condoms in Bangladesh [2,3]. Whereas overall HIV prevalence is less than 1% in most key vulnerable population groups (e.g. sex workers, men who have sex with men, transgenders, migrant workers) but a high prevalence of HIV (5.3%) among injecting drug users(IDUs) in Dhaka has been documented [4-6]. Further, a number of studies consistently reported high levels of preventable and treatable STIs among mobile populations, transport workers, IDUs and sex workers (SW); and more than one third of the sex workers reported sexual contact with IDUs and mobile populations [4-9]. The overlap among “bridging populations” suggests that there is a risk of HIV transmission from one group to other group of vulnerable populations if the epidemic spreads to sex workers (and then from SWs to their clients) [5,6].

Despite limitations of sampling, question design and variations on results, a range of studies consistently show that a high proportion of men in Bangladesh do not use condoms in the majority of their commercial (also non-commercial) sexual encounters [4-6,10-14]. A synthesis of recent studies among diverse groups of vulnerable population (such as migrant taxi drivers, rickshaw pullers, fishermen, Injecting drug users and truckers) suggest that only between 6% and 25% (average 13%) have ever used condoms when buying sex and very few of them even realized that they were at risk of exposure to HIV and other STIs [4-6,10-14]. A number of studies also reported that population like rickshaw pullers, internal migrant workers, truckers and fishermen are the key segment of clients of the SWs and have visited a sex worker on an average 3–4 times in the last month [10-13].

While exploring the questionnaires used by different studies for assessing condom use behavior in Bangladesh, it was noted that although limited work has been done to examine the prevalence of condom use [11-13], very few studies have measured attitudes towards condom using any culturally standardized scale [10]. Thus, actual attitudes and barriers to condom use remain poorly understood. The reliable and valid measurement of attitudes towards condom use may enhance the efforts to design population specific interventions aimed at promoting more positive attitudes and increased use of condoms.

Although a variety of instruments have been used in a number of studies from different settings to measure attitudes towards, and barriers to, condom use with different populations [15-18], the most commonly used instrument to measure attitudes towards condom use in sexual health and HIV research is the ‘Attitude Towards Condoms Scale’ (ATCS), developed in the U.S. [19]. This is a 40-item self-report instrument originally developed base on concepts of the theory of reasoned action [20,21] and as an assessment instrument for use in research directed towards promotion of barrier methods of contraception [15]. However, several studies with men and women had demonstrated the utility of the ATCS scale and provided further support for the criterion related validity of the instrument [15,16]. Brown [19] suggested that a method-specific scale would be more appropriate than a general measure of condom use. Several sexual health/HIV studies have also used 10–15 items from this scale with or without modification in different combinations [22-24]. Brown and Chen [25] recently validated a 4-item scale among rural African-American women. One recent study among migrant workers in Kolkata, India adopted 10 items from this scale to measure attitudes towards condom use among the study population and demonstrated the utility of the short version [26]. Further study among migrant workers in Kolkata, India re-validated a similar 10-item scale and tested out the feasibility of using only 6 items among same study population [27].

It is well known that measures that have demonstrated reliability and validity in one population may not necessarily be generaliseable to other populations. In each new population, it is important to assess an instrument’s cultural relevance and to re-validate it [28]. Recognition of this has led to the concept of ‘cultural equivalence’, which can be defined as a psychological assessment tool’s applicability, reliability and validity with specific group of people who belong to a different cultural group from those with whom the tool was originally assessed [29]. Although Brown’s ATCS scale is widely validated in western world and appropriate for English speaking (and writing) individuals, its utility and cultural applicability in vulnerable groups with low literacy rates has scarcely been evaluated.

It is important for a setting, where very low levels of condom use are consistently reported, to monitor attitudes towards and uptake of condoms on regular basis in order to scale-up HIV and STIs prevention initiatives. As there was no uniform-standardized condom attitude scale for Bangladeshi population, this study translated and cross-culturally adapted items from the English ATCS, and examined the validity and reliability aspects of the Bengali version of ‘attitudes towards condoms use (ATC-B) scale’ to be used in vulnerable population groups in Bangladesh.

## Methods

### Design and participants

This paper reports mainly on the survey components of a mixed-method sexual health research study (later referred as main study throughout this paper) amongst a sub-sample of rural-to-urban migrant workers in Bangladesh. The ATC-B scale validation was carried out as a part of that main study. The cross-sectional survey sample (n = 878) comprised rural-to-urban migrant taxi drivers (n = 437) and restaurant workers (n = 441). The study selected taxi drivers, restaurant workers and female garments workers (n = 1033) since they are considered as important population segments of rural-to-urban migrant workers in Dhaka. However, in order to capture the male perspectives of condom use, the ATC-B scale was not tested out among female garments workers, therefore, excluded from this paper. Here we report specifically on the development, psychometric evaluation and validation of a culturally appropriate short and simplified version of ATC-B scale.

The survey sample was randomly selected using a two-stage cluster sampling approach and comprised sexually active population who had moved from their rural place of origin into urban Dhaka for work purposes. Inclusion criteria were: (i) living in Dhaka for over 1-year whilst maintaining their permanent residence in the rural village (ii) age between 18 and 35 years, (iii) working in Dhaka as either garments worker or taxi driver or restaurant worker, and (iv) capable of independent communication and giving informed consent to this study. Prior to enrollment for the study, the research team members informed the participants about the inclusion criteria and the research protocol including the time commitment during the recruitment process. Written informed consent was obtained before administering the instrument. The study was approved by the institutional ethics review boards (Bangladesh Medical Research Council and the University of Nottingham, United Kingdom).

### Instrument

The ACT-B scale consisted of 10 items and was structured in a way that the questions around condom use were asked first and then about their attitudes towards use. The scale items were structured in three dimensions: condom vs. sexual satisfaction; condom vs. gender dimension; and condom vs. sexual interest.

The 10-item ACT-B scale was nested-in the survey questionnaire of main sexual health research study and tested out along with that questionnaire. The development of questionnaire was informed by findings from a formative study [10]. During the course of the main study, a self-report questionnaire was administered which gathered information about sexual practices, AIDS-related knowledge, perception of HIV transmission and preventive behaviours (e.g. condom use and attitudes towards condom use). A paper detailing the instrument development, sample size, sampling strategy, recruitment and data collection method of the main study has been published elsewhere [10].

Attitudes towards condoms were measured with 10-item Likert-type scale of which six items (those were directly related to current research aim) were adapted from the original ‘Attitudes Toward Condom Use Scale’ [19] (these were also re-validated recently amongst migrant workers in India) [26,27]. The scale is bi-dimensional that includes both positive and negative attitudes. The remaining four items of the scale were developed based on focus group discussions with the participants and cultural perspectives of Bangladesh, and tested out with 6 items taken from the original scale. Each item was rated on a five-point Likert scale (with 1 = strongly disagree; 5 = strongly agree). Thus, the total score for 10-item scale ranged from 10 to 50 (10 = strongly negative; 25 = neither negative nor positive; 50 = strongly positive). Contents and terms for the new short version of ATC-B were constructed based on a review of the literature and focus group data with a small group of migrant workers in Dhaka.

During this study we also invited the participants to attend focus group meetings (total four sessions with 39 participants; two with taxi drivers and two with restaurant workers) to consider and finalize the terms used in the questionnaire measuring attitudes towards condoms. Also, the content validity and cultural equivalency of the terms used in the scale was explored according to Herdman et al.’s [29] criteria and then included in the main questionnaire as a sub-section. This participatory discussion with the study population helped the development process of culturally equivalent translation of the items used in the scale. Along with the questionnaire of the main study, these 10 items were also translated into local language (Bengali) and back translation was done by an independent expert to check the accuracy of translation. The 10-item scale was then incorporated in the main sexual health survey questionnaire for testing out among a selective group of rural-to-urban migrant men (taxi drivers and restaurant workers).

### Data collection

Data collection for the main survey (along with 10-item scale) occurred over a four- month period while data collection for the test-retest component was about two weeks. It is suggested that 10% of the subjects who have completed the initial survey are sufficient to estimate the reliability of a measure [30]. We obtained mobile phone numbers of 878 participants those who completed the initial questionnaire. Of them 175 (20%) agreed to complete the instrument for a second time, whom we contacted two weeks after they completed the initial survey to retake the questionnaire to evaluate its test-retest reliability (repeatability). A unique identifier was allocated for each participant and was matched with contact information of the individuals; which facilitated tracing potential participants for the second survey. A total of 114 participants finally participated in the second survey.

### Statistical analysis for validation of scale

Quantitative data were analyzed using the Statistical Package for Social Sciences (SPSS) version 17.0. Pairwise procedures were applied to handle missing data; that is, variables with missing values were excluded pairwise from the analysis. Descriptive statistical information (e.g. means, proportion, and variability) was examined for all variables. Pearson’s and Point-biserial correlations were used to examine the scale items in terms of their mean response scores and the correlation matrix between items, and to assess relationships between two continuous variables (e.g. condom attitudes and age), and relationships between continuous and dichotomous variables (e.g. condom attitudes and condom use). Researchers suggested that in order to perform more precision in measurement, it is appropriate to sum the individual item score on each item of a Likert type scale to produce an interval-level total scale variable [31-33]. Cronbach’s alpha was computed using all individual items as data. The two-way mixed model (single measure) Intra-class correlation coefficient (ICC) was calculated to evaluate the test-retest reliability (repeatability). A 95% confidence interval was used to describe the variety/differences in the ICCs. The test-retest (repeatability) procedures were conducted before the factor analysis procedures.

Principal components factor analyses were computed to study the underlying structure and estimate the construct validity of the ATC-B scale and ‘condom use with different partners’ variable, testing the significance in the difference between the users and non-user group. Exploratory factor analysis procedures with varimax rotation were conducted to confirm its unifactorial structure. In this study, items were retained only if the factor loading was above 0.40. In addition, only components with Eigen values greater than 1.00 were retained for the interpretation. The principal component analysis was conducted using case-wise deletion. After reverse-scoring the negative attitudinal items, all items were summed for an overall measure, in which the higher scores represented more positive attitudes toward condoms (possible range = 10–50).

### Qualitative data analysis

Qualitative data were translated and transcribed verbatim, and analyzed thematically. Extensive discussions were held between the members of researcher team to explore the key themes. On the basis of these discussions, we developed coding matrix for thematic analysis and identified emerging themes in the data set. We further analyzed data based on Herdman et al.’s [29] criteria for achieving conceptual and cultural equivalency of the scale. This rigorous process allowed us to identify key contents and themes where appropriate. Finally, qualitative data was organized, and the central concepts were described.

## Results

### Characteristics of the survey respondents

Table 1 presents selected characteristics of the survey respondents. The results suggest that 49.7% of the participants were taxi drivers and 50.3% were restaurant workers. Further, 69% of the respondents were married and 86% had migrated from rural villages. The average monthly income of the participants was Taka 4796 (approximately US$ 58). Among those who were married (n = 601), 56% were currently living without their spouse in Dhaka due to work migration. The participants reported access to some form of mass media (radio or television). Nearly a quarter of the respondents (24%) did not have any exposure to media or campaign sources. Over one-half of participants used drugs (mostly cannabis) or drank alcohol. Only a few respondents (7%) had ever injected drugs. Nearly half of the men (48%) had paid for sex in the past month, and 48% reported unprotected paid sex in the past 3 months. A high proportion of the respondent was not aware of the risk associated with unprotected sex (87%) or injecting drugs (75%). Although 75% (n = 659) of the respondents (n = 878) had ‘ever heard’ about HIV and AIDS, only one-third (36%) perceived themselves to be at risk of getting HIV or other STIs. Very few respondents used condoms during the last paid sexual encounters and only 12% know that condom can protect against STIs including HIV.

**Table 1:** Distribution of survey respondents by background characteristics

**Variables**	**Taxi drivers n = 437(%)**	**Restaurant workers n = 441 (%)**	**Total n = 878 (%)**
**Current age (Mean)**	(28.3)	(28.3)	(27.4)
18-24 years	105 (24%)	111 (25.2%)	216 (24.6%)
25-30 years	216 (49.4%)	221 (50.1%)	437 (49.8%)
31-35 years	116 (26.5%)	109 (24.7%)	225 (25.6%)
**Education**			
No education	100 (22.9%)	118 (24.3%)	201 (22.9%)
Primary (level I-V)	182 (41.6%)	168 (38.1%)	367 (41.6%)
Secondary (level VI-X)	155 (35.5%)	155 (37.6%)	310 (35.5%)
Income/month in Bangladeshi Taka*	5737.9	3853.4	4795.7
**Migrated from**			
Town/sub-district	57 (13%)	65 (14.7%)	122 (13.9%)
Village/rural	380 (87%)	376 (85.3%)	756 (86.1%)
**Marital status**			
Married	296 (67.7%)	305 (69.2%)	601 (68.5%)
Never-married	141 (32.3%)	136 (30.8%)	277(31.5%)
**Current living status†**			
Lives with spouse/family	127 (29%)	135 (30.6%)	262 (29.8%)
Lives alone	310 (71%)	306 (69.4%)	616 (70.2%)
**Used cannabis like drug in past 3 months**	247 (56.5%)	268 (60.8%)	515 (58.7%)
**Drank alcohol as recreational habit in 3 months**	258 (59.0%)	267 (60.5%)	525 (59.8%)
**Injected drugs in past year**	24 (5.5%)	38 (8.6%)	62 (7.1%)
**Age at sexual debut (Mean)**	(17.65)	(16.89)	(17.67)
**BCC exposure**			
No Exposure	104 (23.8%)	119 (27.0%)	223 (24.5%)
Exposed to one message type	181 (41.4%)	186 (42.2%)	367 (41.8%)
Exposed to multiple message types	152 (34.8%)	136 (30.8%)	288 (32.8%)
**Had paid sex in the last month**	212 (48.5%)	205 (46.5%)	417 (47.5)
**Had unprotected paid sex in the past 3 months**	265 (60.6%)	243 (55.1%)	508 (57.9%)
**Perception of risk & knowledge of HIV/AIDS**			
Ever heard of HIV/AIDS	322 (73.7%)	337 (76.4%)	659 (75.1%)
Ever heard about VCT services	79 (18.1%)	67 (15.2%)	148 (16.9%)
Perceived themselves at risk of HIV/STIs	161(36.8%)	154(34.9%)	315(35.9%)
Aware of risk of unprotected sex	55 (12.5%)	57 (12.9%)	112 (12.8%)
Aware of risk of injecting	104 (23.8%)	112 (25.4%)	216 (24.6%)
Knows that condom can protect against HIV/STIs	50 (11.6%)	57 (12.9%)	107 (12.2%)
Used a condom at last paid sex	61 (21.8%)	73 (24.5%)	134 (23.2%)
Ever used a condom to avoid STIs or HIV/AIDS	126 (29.5%)	137 (31.1%)	263 (30.0%)

NA = not applicable; BCC, Behavior Change Communication; VCT, voluntary counseling and testing;

*1 US$ = Taka 84, †Married respondents only.

### Response with respect to scale items

In general, respondents reported negative attitudes (or being indifferent about condom use) toward use of condoms; the mean of the condom attitude items was 21.45 (*SD* = 6.78) on a scale with a possible range of 10–50 (10 = strongly negative attitude; 25 = neither negative nor positive attitude; 50 = strongly positive attitude). About 68% of the respondents strongly disagreed/disagreed with the statement ‘proper use of condoms enhances sexual pleasure’. Similarly, 77% strongly agreed/agreed that ‘using condoms are unmanly’.

Table 2 illustrates item wise association between condom attitudes and proportion of condom users. For the ease of this analysis, responses on five-point Likert scale were collapsed in to three groups; e.g. disagreed (1–2), neither agreed nor disagreed (undecided/neutral) (3) and agreed (4–5). In general, analysis across all 10 items revealed that the participants with strong negative attitude towards condom use were significantly less likely to use condoms.

**Table 2:** Item wise association between condom attitudes and condom use (n = 878)

**No.**	**Items - condom attitudes**	**# In group (n = 878)**	**% Used condom**	**P value**
1	Condoms are uncomfortable			0.000
	Strongly disagreed/Disagreed	235	67.7	
	Undecided/neutral	102	42.9	
	Strongly agreed/Agreed	541	14.3	
2	The idea of using condoms does not appeal to me			0.003
	Strongly disagreed/Disagreed	238	52.8	
	Undecided/neutral	104	33.3	
	Strongly agreed/Agreed	536	20.3	
3	Using condoms make sex un-enjoyable			0.000
	Strongly disagreed/Disagreed	236	63.9	
	Undecided/neutral	106	25.0	
	Strongly agreed/Agreed	536	19.6	
4	Proper use of condoms enhance sexual pleasure			0.000
	Strongly disagreed/Disagreed	535	11.0	
	Undecided/neutral	163	41.7	
	Agreed	180	75.0	
5	I would avoid using condom if possible			0.011
	Strongly disagreed/Disagreed	178	45.5	
	Undecided/neutral	132	52.6	
	Strongly agreed/Agreed	568	22.6	
6	I just don’t like the idea of using condoms			0.000
	Strongly disagreed/Disagreed	205	76.7	
	Undecided/neutral	132	38.1	
	Strongly agreed/Agreed	541	10.8	
7	Men who use condoms show concern and responsibility to their partner(s)			0.000
	Strongly disagreed/Disagreed	348	16.2	
	Undecided/neutral	307	40.0	
	Strongly agreed/Agreed	233	58.3	
8	Using condoms are unmanly			0.012
	Strongly disagreed/Disagreed	179	45.0	
	Undecided/neutral	128	56.3	
	Strongly agreed/Agreed	571	23.6	
9	Condoms are the best way to protect myself from HIV and against other STIs			0.000
	Strongly disagreed/Disagreed	530	13.4	
	Undecided/neutral	118	41.9	
	Strongly agreed/Agreed	230	59.3	
10	Suggestion from a partner to use a condom mean that she doesn’t trust her partner			0.000
	Strongly disagreed/Disagreed	212	66.7	
	Undecided/neutral	128	37.5	
	Strongly agreed/Agreed	538	12.3	

Items 1, 2, 3, 5, 6, 8 and 10 are scored negatively. Items 4, 7 and 9 are scored positively.

### Relationships between condom attitudes, condom use and sexual risk behaviors

We examined correlations of condom attitudes with selected socio-demographic factors and HIV-related risk behaviors. The respondents who were married (r = 0.158, p < 0.05), higher levels of education (r = 0.087, p > 0.05), listened to radio/television on regular basis (r = 0.076, p > 0.05) and accurate knowledge of HIV transmission and prevention (r = 0.173, p < 0.05) were more likely to report positive attitudes towards condoms. Alternatively, participants who reported high levels of risky sexual behaviors (r = −0.172, p < 0.05), having had more than one paid sex partners (r = −0.159, p < 0.05) or never had or inconsistently used condoms (r = −0.384, p < 0.01) were more likely to hold negative attitudes about condoms. In addition, they were more likely to have paid sex without using a condom in the past three months and have had low perceived risk of HIV. The participants who reported having had commercial sex without using a condom in the last 3 months were displayed high negative attitude towards condom (r = −0.688, p < 0.001).

Further analyses indicated different patterns of condom attitude and relationships by type. Among married men with multiple partners in the past 12 months, analyses indicated that absence from spouse due to work-migration and low perception of risk were related to more negative attitudes toward condoms (r = −0.217, p < .01 and r = −0.163, p < .05 respectively).

### Reliability analysis of the scale

The result of the test-retest reliability (repeatability) is shown in Table 3. The internal consistency (Cornbach’s alpha, *α*) of the scale was 0.89. Overall, the 2-week ICC values ranged from 0.62 to 0.77, with the lowest value recorded for “would avoid using condom if possible” and the highest value for “condoms are uncomfortable”. The majority of the ICC values were found to be above the recommended value of 0.2 [15,34] and were significant for all items on ATC-B (p < 0.001). The 2-week test-retest reliability (repeatability) of the ATC-B was also assessed by the Pearson product–moment correlation coefficient with a value of 0.75.

**Table 3:** Test-retest reliability based on intra-class correlation coefficient for the ATC-B (N = 114)

	**ATC-B items**	**ICC Value**	**95% CI**
1	Condoms are uncomfortable	0.77	0.58–0.86
2	The idea of using condoms does not appeal to me	0.71	0.53–0.82
3	Using condoms make sex un-enjoyable	0.74	0.49–0.78
4	Proper use of condoms enhance sexual pleasure	0.76	0.51–0.85
5	Would avoid using condom if possible	0.62	0.47–0.72
6	I just don’t like the idea of using condoms	0.70	0.48–0.79
7	Men who use condoms show concern and responsibility to their partner(s)	0.73	0.50–0.82
8	Using condoms are unmanly	0.68	0.48–0.77
9	Condoms are the best way to protect myself from HIV and against other STIs	0.69	0.49–0.79
10	Suggestion from a partner to use a condom mean that she doesn’t trust her partner	0.76	0.57–0.87

ATC-B; attitudes toward condoms – Bengali Version; ICC; intra-class correlation coefficient (single measure).

### The scale structure and validity analysis

Construct validity procedures were used to determine “how well an instrument measures and what it is supposed to measure” [35]. The procedures of principal component factor analysis with varimax rotation and item-subtotal correlations were performed to assess the construct validity of the ATC-B (see Table 4). Principal component analysis of these 10 items yielded two principal components. The first component accounted for about 52% of the variance, with second component about 20% of the total variance. The Eigen-value for both factors was greater than one (Table 4).

**Table 4:** Summary of principal component analysis of 10-item condom attitude scale (n = 878)

**Component**	**Initial Eigen values**			**Extraction sums of squared loadings**		
	**Total**	**% of Variance**	**Cumulative%**	**Total**	**% of Variance**	**Cumulative %**
1	6.159	61.585	61.585	6.159	61.585	61.585
2	2.019	20.191	81.776	2.019	20.191	81.776
3	.426	4.260	86.036	.426	4.260	86.036
4	.338	3.380	89.416			
5	.323	3.231	92.647			
6	.245	2.449	95.096			
7	.184	1.840	96.936			
8	.150	1.500	98.436			
9	.093	.930	99.366			
10	.063	.634	100.000			

Items 1, 2, 3, 5, 6, 8 and 10 are scored negatively. Items 4, 7 and 9 are scored positively.

Extraction Method: Principal Component Analysis.

The Eigen-value for factor three was less than one. The application of scree test also suggests only two factors was dominant (Table 4). Furthermore, the analysis revealed that all items were strongly loaded in component 1, which explains maximum variance (Tables 4 and 5). Only item ‘would avoid using condom if possible’ had more positive loading with component 2. Therefore, if we reject this item, remaining 9-item scale could be considered as unifactorial scale for measuring condom attitude for further study among vulnerable populations.

**Table 5:** Component matrix of 10-item condom attitudes scale (n = 878)

**No.**	**Items**	**Component**	
		**1**	**2**
1	Condoms are uncomfortable	.873	-.380
2	The idea of using condoms does not appeal to me	.768	-.412
3	Using condoms make sex un-enjoyable	.831	-.331
4	Proper use of condoms enhance sexual pleasure	.874	.198
5	Would avoid using condom if possible	.668	.542
6	I just don’t like the idea of using condoms	.787	-.08
7	Men who use condoms show concern and responsibility to their partner(s)	.846	.096
8	Using condoms are unmanly	.754	-.094
9	Condoms are the best way to protect myself from HIV and against other STIs	.770	.0163
10	Suggestion from a partner to use a condom mean that she doesn’t trust her partner	.876	-.234

Items 1, 2, 3, 5, 6, 8 and 10 are scored negatively. Items 4, 7 and 9 are scored positively.

Further, the differences in mean scores of 10-item scale between condom-user and never-user group were also compared. The mean score of the condom user group was 23.64 and that of the never user group was 18.58. This difference in mean scale score was statistically significant (p < 0.01).

### Content validity and cultural equivalency

The focus group participants went on to consider the Bengali written version of the ACT-B scale. Based on Herdman et al.’s [29] criteria for achieving conceptual and cultural item equivalence, we found that the items used in the ACT-B were simple and easily understood to most of the focus group participants once they were translated into Bengali. During focus group discussions with restaurant workers and taxi drivers most of the participants reported that the meaning of the ACT-B questions was often very clear to them. As one of the restaurant worker expressed his understanding with the ACT-B questionnaire as follows,

“The questionnaire looks fine with me. The questions are very simple and easy to understand”.

(24-year old restaurant worker in focus group discussion)

In terms of item equivalence, or the items on the scale being relevant and acceptable to the respondents, most of the participants from both groups reported that they clearly understood the Bengali terms and contents used in the questionnaire. The Bengali phrases or words used in the questions of the ACT-B were considered to be simple by the taxi drivers and restaurant workers. Also, the meaning of the questions was considered clear to most of the respondents participated in the focus groups. During the focus groups with the taxi drivers the participants frequently highlighted the greater clarity of translated Bengali ACT-B scale. As one of the taxi driver from this group described,

“I can’t see any problem understanding the meaning of the contents used in the scale. You used very simple terms”.

(32-year old taxi driver in focus group discussion)

## Discussion

The results of this study indicate that ATC-B had acceptable metric properties for assessing attitudes toward condom use in vulnerable populations. The study aim to develop and test the content validity and reliability of ATC-B scale for vulnerable populations was achieved. While other ‘attitudes toward condom use scales exist; tested brief scale was found reliable. This scale is bi-dimensional and captured both positive and negative attitudes toward condom use as well as the benefits of using condoms.

Our research has also shown that it is possible to develop culturally acceptable mode of measuring attitudes towards condom use in a highly selective population. We considered our scale according to Herdman et al.’s [29] criteria for achieving cultural item equivalence. The result suggests that the items used in the ACT-B scale were relevant and easily understood once they were translated into Bengali. This method of developing and validating culturally appropriate scale could also be applied to other settings, survey tools or other vulnerable groups.

To determine the number of principal components, only components with Eigen values greater than 1.00 were retained for interpretation. This criterion is best used when principal component analysis is used as a technique to extract components [31,34,36]. The factor analysis provides us a one-factor solution. Based on the above findings this scale satisfies the criteria of – high construct validity, internal consistency and high inter-item correlations. These findings are consistent with previous studies that have validated short forms of condom attitude scales in a range of populations and settings [25-27]. The 2-week test-retest reliability (repeatability) of the ATC-B demonstrated that the scale had acceptable consistency over time [35-37]. The repeatability of the ACT-B as evidenced by substantial agreement for most of the items was similar to those of other studies [19]. Overall the reliability coefficients for all items on the ATC-B indicate that the instrument is reproducible and internally consistent and is comparable to the findings in previous studies [19].

Although the average sum of the condom attitude scale items (score = 21.45 ± 6.78) was deviated towards negative pole, such mean score close to neutral cut-off point (25 = neither negative nor positive attitude) may be otherwise suggestive of being indifferent about condom use by the sample. Given that a vast majority of the sample did not use condoms in commercial sexual encounters in Bangladesh, the study suggests a need to reinforce prevention efforts through promoting consistent and effective use of condoms in high-risk settings. However, in terms of condom use, it may be that a more positive attitude toward condoms must occur before the use of this particular device is readily accepted by men. The ATC-B scale appears to be a psychometrically valid instrument for measuring the attitude towards condoms and can be of use to monitor progress of the promotional activities focusing the actual use of condoms.

Although several limitations (cross-sectional study design, modest sample size, highly selective sample etc.) were inherent in the study design, the development and testing of this scale adds to the existing body of knowledge about the use of condoms as well as demonstrating the validity and reliability of this brief ATC-B scale in the Bangladeshi context.

There might be a possibility of measurement error as data was collected as part of a larger study which measured sexual behavior, condom use behavior and tested condom attitudes scale at the same time. Moreover, our study population was highly selective and a sub-sample of rural-to-urban migrant workers in Dhaka. This may limit the generalisability of this study results to other populations. However, research does indicate that our study population forms a crucial part of the high-risk populations in Bangladesh, especially as the clients of sex workers [10-14]. In spite of these limitations, the ATC-B scale tested out in this study is a short, reliable and culturally-standardized instrument to measure attitudes towards condom use.

## Conclusion

The ATC-B scale demonstrated satisfactory construct validity and internal consistency reliability in this sub-sample of rural-to-urban male migrant workers in Bangladesh. The process by which the items were generated and selected by the study participants themselves, adds to the cultural relevance and geographical specificity of the scale. The findings support further use of this scale in prevention initiatives targeting vulnerable populations for HIV and STIs in Bangladesh in order to monitor condom uptake and the progress of condom promotion campaigns and research in similar groups.

## Competing interests

We declare that we have no competing interests.

## Authors’ contributions

TR conceptualized and designed the study. TR, MSR & MR conducted the statistical analyses. All authors made significant contributions to the conception and design of the analyses, interpretation of the data, and drafting of the manuscript, and all authors approved the final manuscript.

## Pre-publication history

The pre-publication history for this paper can be accessed here:

http://www.biomedcentral.com/1471-2458/13/240/prepub
